# Polyps’ Extension and Recurrence in Different Endotypes of Chronic Rhinosinusitis: A Series of 449 Consecutive Patients

**DOI:** 10.3390/jcm13041125

**Published:** 2024-02-16

**Authors:** Leonardo Calvanese, Cristoforo Fabbris, Giuseppe Brescia, Valerio Maria Di Pasquale Fiasca, Alessandra Deretti, Francesco Finozzi, Leonardo Franz, Anna Chiara Frigo, Gino Marioni

**Affiliations:** 1 ENT Unit, Department of Surgery, Ospedali Riuniti Padova Sud, 35043 Monselice, Italy; leonardo.calvanese@aulss6.veneto.it (L.C.); cristoforo.fabbris@unipd.it (C.F.); 2 Department of Medicine DIMED, Padova University, 35100 Padova, Italy; 3 Section of Otolaryngology, Department of Neuroscience DNS, Padova University, 35100 Padova, Italy; valerio.fiascadp@gmail.com (V.M.D.P.F.); alessandra.deretti@aopd.veneto.it (A.D.); francescofinozzi@gmail.com (F.F.); 4 Phoniatrics and Audiology Unit, Department of Neuroscience DNS, Padova University, 31100 Treviso, Italy; leonardo.franz@unipd.it (L.F.); gino.marioni@unipd.it (G.M.); 5 Department of Cardiac-Thoracic-Vascular Sciences and Public Health, Padova University, 35100 Padova, Italy; annachiara.frigo@unipd.it

**Keywords:** CRSwNP, endotypes, asthma, ASA intolerance, eosinophils, basophils, ESS

## Abstract

Different inflammatory endotypes reflect the heterogeneity of chronic rhinosinusitis with nasal polyps’ (CRSwNPs) clinical presentation. This retrospective study aimed to analyze the distribution of polyps in nasal cavities and paranasal sinuses to establish a possible association between CRSwNP endotypes, prognosis, and polyps’ extension. This study included 449 adult patients who underwent endoscopic sinus surgery for CRSwNPs between 2009 and 2022. Patients were categorized based on the number of paranasal sinuses involved by polyps. Statistical analyses, including Cox regression, were performed to identify associations between demographic, clinical, and histopathological factors and disease recurrence. CRSwNP patients were stratified into four groups based on the extent of polyp involvement. Asthma and acetylsalicylic acid (ASA) sensitivity were associated with more sinuses involved (*p*-values = 0.0003 and 0.0037, respectively). Blood eosinophil counts increased with the number of sinuses affected (*p*-value < 0.0001). The distribution of eosinophilic and non-eosinophilic histotypes varied significantly among these groups (*p*-value < 0.0001). The risk of CRSwNP recurrence was higher in patients with asthma, higher basophil percentages, and eosinophilic histotype (*p*-value 0.0104, 0.0001, 0.0118, and 0.0104, respectively). This study suggests a positive association between the number of paranasal sinuses involved by polyps and the severity of CRSwNPs, particularly in patients with eosinophilic histotype, asthma, and ASA sensitivity.

## 1. Introduction

Chronic rhinosinusitis (CRS) was defined in the European Position Paper on Rhinosinusitis and Nasal Polyps in 2020 [1] as an inflammatory disease of the nose and paranasal sinuses lasting more than 12 weeks. Estimating the prevalence of CRS is challenging as different studies report varying results. However, it affects more than 31 million people annually in the United States and is characterized by symptoms that impact the quality of life, mainly nasal obstruction, and loss of smell [2,3]. Being a chronic disease, it determines a considerable annual cost per patient [1,4].

Traditionally, CRS is divided into two phenotypes: CRS with nasal polyps (CRSwNPs) and without nasal polyps (CRSsNPs). Polyps’ presence accounts for 20% of CRS [5,6].

The heterogeneity of the clinical presentation of CRSwNPs is reflected by a variety of inflammatory endotypes. Histological analysis of CRS surgical specimens shows the presence of an immunity cell infiltration of different species [7]. Depending on the most represented type of leukocytes, CRSwNP is pathologically divided into eosinophilic (eCRSwNP) and non-eosinophilic (non-eCRSwNP) subtypes [8,9,10,11]. The eCRSwNP subtype is associated with a more severe endoscopic pattern [12] and a higher polyps’ relapse rate after treatment [7,13,14,15]. According to previous studies, indeed, eosinophilic inflammation with neutrophil-related markers was associated with disease extent [1]. Similarly, asthma-associated CRSwNPs is considered more aggressive in terms of resistance to medical therapy and related to a higher recurrence rate after endoscopic sinus surgery (ESS) [16,17]. Among eCRSwNPs, aspirin-exacerbated respiratory disease (AERD) is represented by eosinophilic asthma and respiratory reactions to cyclooxygenase-1 (COX-1) inhibitors [18]. A poorer overall prognosis (in terms of polyps’ recurrence) has been described in patients affected by AERD [19,20,21,22,23,24]. In the case of failure of local and systemic medical therapy, the current treatment of CRS involves ESS consisting of a modular approach including all or some of the following procedures: anterior and posterior ethmoidectomy, middle antrostomy, sphenoidotomy, and frontal sinus approach [25].

In 2005, Andrews et al. [26] investigated the most frequent sites of origin of nasal polyps; they found that 97.4% originated from the anterior ethmoid complex and middle meatus. On the other hand, they reported that maxillary and frontal sinuses were less frequently involved; sphenoidal sinus involvement was unusual. The reason for this distribution has yet to be definitively explained. To the best of our knowledge, no available studies have investigated the relationship between the intra-operative extension of sinonasal polyps and CRSwNP endotypes. This retrospective study aimed to analyze the intra-operative distribution of polyps in nasal cavities and paranasal sinuses in order to establish a possible association between CRSwNP endotypes and disease recurrence risk.

## 2. Materials and Methods

This study was conducted in accordance with the principles of the Helsinki Declaration. All patients signed a detailed informed consent form and gave their written permission for clinical case publication. Data were examined in accordance with Italian privacy and sensitive data laws and the in-house regulations of Padova University’s Otolaryngology Section.

A consecutive series of 486 patients (14–84 years old) who had undergone ESS for CRSwNPs at the Otolaryngology Section of Padova University between 2009 and 2022 was retrospectively considered. The inclusion criteria for patients’ recruitment were the following: confirmed diagnosis of CRSwNPs by endoscopy and radiological evaluations; primary surgical treatment for CRSwNPs; final postoperative histological diagnosis of CRSwNPs. All surgical procedures were performed by the same surgeon (G.B.). Patients affected by autoimmune disorders, other inflammatory or hematological disorders, malignant neoplasms, acute or chronic infectious diseases other than sinusitis, allergic fungal rhinosinusitis, and conditions that only affected one side of the sinuses (such as fungus ball, antrochoanal polyp, and sinusitis related to odontogenic etiology) were ruled out. Finally, 449 patients met the inclusion criteria and were enrolled in this retrospective study.

Patients’ clinical histories of asthma and allergies, including sensitivity to acetylsalicylic acid (ASA) or other non-steroidal anti-inflammatory drugs, were obtained from their clinical charts. Asthma diagnosis was based on reversible signs of obstruction during methacholine challenge spirometry, according to the definition of the Global Initiative on Asthma [27].

Each patient underwent ESS under general anesthesia with ethmoidectomy, middle antrostomy, sphenoidotomy, and/or frontal sinusotomy, depending on involved sites, with septoplasty and turbinoplasty, if necessary. Data were collected from surgical reports regarding the location of nasal polyps during ESS. Patients with involvement of only one paranasal sinus, unilateral or bilateral, were included in group 1. Those with involvement of 2, 3, or 4 paranasal sinuses, unilateral or bilateral, were included in groups 2, 3, and 4, respectively. Patients with polyps occupying only the nasal cavities were associated with group 1.

All patients were treated post-operatively with isotonic saline solution irrigations twice a day (20 mL per irrigation) and nasal steroids [fluticasone furoate 110 μg daily (55 μg per nostril) or mometasone furoate 200 μg daily (100 μg per nostril)]. Adequate therapy was prescribed for asthmatic and allergic patients. Follow-ups with rigid 0° or 30° endoscopes were scheduled at 3, 6, and 12 months after ESS and yearly thereafter.

For all patients considered in this study, the following variables were collected: preoperative blood eosinophils and basophils cell counts and percentages, polyps’ location as found during ESS, histological type, and recurrence-free interval. Patients were classified as recurrent if they developed endoscopic evidence of at least grade I polyposis according to nasal polyp score classification during follow-up [28].

### 2.1. Histopathological Investigations

Histopathological investigation was performed by the same dedicated pathologist.

Surgical tissue samples were fixed in 10% buffered formalin, embedded in paraffin, cut into 4 μm thick sections, and routinely stained with hematoxylin and eosin (H&E). Every H&E slide was examined under a light microscope at low-power magnification (40×) to identify the most representative fields for each histological feature considered. Then, selected areas were examined at high-power magnification (100× or 400×).

The eosinophil count was measured by examining 5 high-power fields (HPFs) (400×) selected from each sample and recording the average number of eosinophils. The eosinophilic histotype corresponded to a mean score of ≥10 eosinophils/HPF. Patients with a mean tissue eosinophil count < 10/HPF were considered to be histologically non-eosinophilic [8,22].

The histological type of the CRSwNPs was analyzed: 207 cases of eosinophilic CRSwNPs (eCRSwNPs) and 242 cases of non-eCRSwNPs were diagnosed.

### 2.2. Laboratory Investigations

For all patients considered in this study, preoperative blood eosinophil and basophil cell counts and percentages were collected. The laboratory tests were performed at least three months after withdrawing oral steroids and one month after withdrawing nasal steroids. They were all processed at the same laboratory (Laboratory Medicine Service, Padova University Hospital), certified in compliance with ISO standard 15189.

### 2.3. Statistical Analysis

The statistical analysis was performed with SAS 9.4 for Windows (SAS Institute Inc., Cary, NC, USA). The results are reported as mean and standard deviation (SD), median and range for quantitative variables, and count and percentage for categorical variables. The significance level was set at the 5% level if not otherwise stated.

The association between the groups identified according to the number of involved sinuses and the patient’s demographic, clinical, and histopathological characteristics was tested with Analysis of Variance (ANOVA) or Kruskal–Wallis test in the case of quantitative variables normally or not normally distributed, with chi-square test in the case of categorical variables. Normality of quantitative variables was graphically inspected with Q-Q plot of residuals obtained from an ANOVA model.

For the pairwise comparisons between groups, the 95% confidence interval (CI) for the difference between means (ANOVA CI), medians (asymptotic CI limits for Hodges–Lehmann estimate), or proportions (Wald asymptotic CI limits) were estimated.

The recurrence-free interval was measured as the time from ESS to recurrence or to last follow-up evaluation for censored patients.

The prognostic role of patients’ demographic, clinical, and histopathological characteristics on recurrence-free interval was assessed with univariate Cox regression.

The proportionality assumption of the Cox regression was checked through the plot of the cumulative Martingale residuals against the values of the covariate and Kolmogorov-type supremum test based on a sample of 1000 simulated residual patterns.

The potential predictors of recurrence found to be statistically significant (*p* < 0.10) at the univariate analysis were entered in a multivariable Cox regression model if not collinear, and the backward stepwise selection was applied. A *p*-value < 0.05 was considered indicative of statistical significance.

The results of the Cox regression have been expressed as *p*-value and hazard ratio (HR) with 95% CI. For quantitative variables, the HR is to be interpreted per 1 unit of increase, except for basophilic blood cell absolute count, where the interpretation should be per 0.01 unit of increase.

## 3. Results

Among 449 enrolled patients (Table 1a), 290 (64.6%) were males, and the mean age was 50.4 years. Patients were stratified into four groups according to the number of sites involved by sinonasal polyps, as described in the Materials and Methods section. Group 1 included 61 patients (37 males), Group 2 included 148 patients (96 males), Group 3 included 123 patients (80 males), and Group 4 included 117 (77 males). In all groups, age and sex distributions were consistent with the distribution in the general population of this study (*p* = 0.4905 and *p* = 0.9178, respectively).

Comorbidities such as asthma and ASA intolerance were found to be associated with the number of sites involved (Table 1a,b). The groups with a higher number of involved sinuses had a higher prevalence of asthma (*p* = 0.0003) and ASA intolerance (*p* = 0.0037). The higher the number of sinuses involved, the higher the blood eosinophils cell percentage and absolute count (*p* < 0.0001 for both). By analyzing polyps’ histological type, the distribution of patients was significantly different among the four groups (*p* < 0.0001). Eosinophilic histotype was most prevalent in groups 3 and 4. The number of paranasal sites involved by polyps was not found to be associated with the patient’s sex, age, allergies, or preoperative blood basophil cell count and percentage.

The effect of demographic, clinical, and histopathological features on nasal polyps’ recurrence and localization was also analyzed (Table 2). At the univariate analysis, the risk of recurrence increased with the number of paranasal sinuses involved (*p* = 0.0073). Moreover, a higher risk of recurrence was associated with asthma (HR = 2.552, 95% CI 1.743–3.738, *p* < 0.0001), ASA intolerance (HR = 2.460, 95% CI 1.569–3.856, *p* < 0.0001), eosinophilic blood cell percentage (HR = 1.054, 95% CI 1.025–1.083, *p* = 0.0002), basophilic blood cell absolute count (HR = 1.108, 95% CI 1.044–1.176, *p* = 0.0007) and percentage (HR = 2.323, 95% CI 1.361–3.962, *p* = 0.0020), and eosinophilic histotype (HR = 2.207, 95% CI 1.480–3.292, *p* = 0.0001. The patient’s sex, age, allergies, and preoperative blood eosinophil counts were not related to recurrence.

Multivariate Cox regression analysis was performed for variables whose association with recurrence had a *p*-value < 0.1 and were not collinear (polyps’ histological type, number of paranasal sites involved by polyps, allergy, asthma, ASA intolerance, percentage count for eosinophilic and basophilic blood cell) applying the backward stepwise selection. The resulting HRs were as follows: asthma (HR = 2.180, 95% CI 1.468–3.238, *p*= 0.0001), blood basophil cells percentage (HR = 2.003 95% CI 1.167–3.439, *p* = 0.0118), and eosinophilic histotype (HR = 1.729, 95% CI 1.138–2.627, *p* = 0.0104).

Data regarding recurrence were missing for 7 out of 449 patients, while preoperative blood eosinophil and basophil values were missing for 1 patient. Statistical analysis regarding disease-free interval was performed for 442 patients. Blood laboratory test values were analyzed regarding polyps’ distribution (groups) in 448 cases and regarding CRSwNP recurrence in 441 cases.

**Figure 1:**
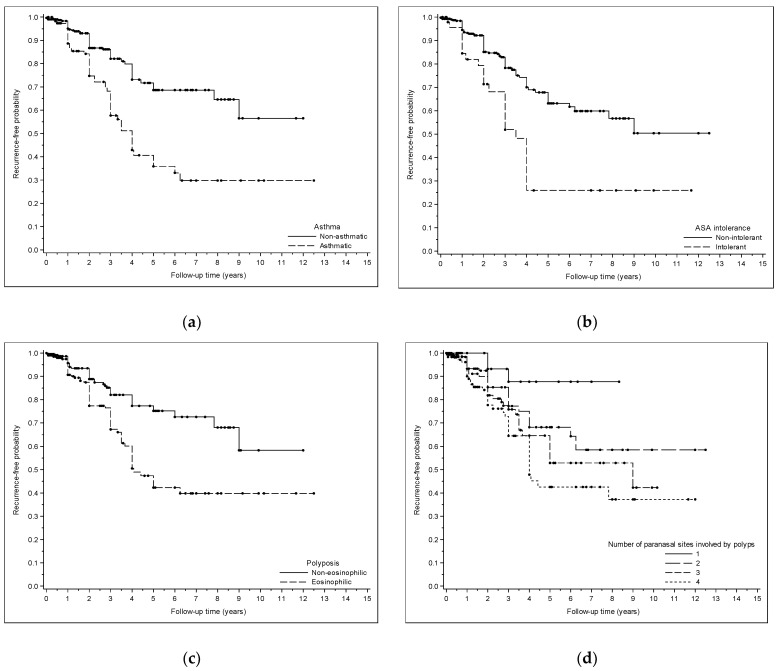
(**a**) Kaplan–Meyer curve showing polyposis recurrence-free probability after surgery in asthmatic and non-asthmatic patients. (**b**) Kaplan–Meyer curve showing polyposis recurrence-free probability after surgery in patients affected and non-affected by ASA intolerance. (**c**) Kaplan–Meyer curve showing polyposis recurrence-free probability after surgery in patients with eosinophilic and non-eosinophilic polyposis. (**d**) Kaplan–Meyer curve showing polyposis recurrence-free probability after surgery in patients with different numbers of paranasal sites involved by polyps.

All results are extensively reported in Table 1 and Table 2. Polyposis recurrence-free probability is graphically represented in Figure 1a–d.

## 4. Discussion

### 4.1. Nasal Polyps’ Extension

This study, based on a large series of patients with CRSwNPs, found a significant association between polyps’ extension and a more aggressive form of eosinophilic polyposis, ASA intolerance, and asthma. Patients suffering from the above-mentioned conditions showed more sinonasal sites involved by polyps. They were preferentially distributed in Groups 3 and 4, as characterized by the highest number of nasal sites involved by polyps.

Patients in the four groups presented an upward trend and an increasing association between the extension of the CRSwNPs and the blood eosinophilia in both cell count and percentage. This link seemed to become more relevant as the disease’s extension increased from group 1 to group 4. If the association between a more extended pattern of CRS and higher values of blood eosinophils is confirmed, the evaluation of those cells could be used to predict the intra-operative degree of the involvement of nasal sinuses. A previous study [29] investigating the association between circulating eosinophil values and the radiological extent of disease showed that a significant increase in circulating eosinophils (>0.24 cells 10^9^/L) was associated with higher total Lund–McKay scores (LMS), particularly concerning maxillary sinus and ethmoidal cells involvement. Furthermore, a significant positive association was found between LMS and the presence of asthma and AERD [30]. From a radiological viewpoint, this agrees with the present study, as our findings intraoperatively demonstrated that, for patients with the aforementioned conditions, nasal polyps extended to more sinuses (Groups 3 and 4). The association between radiological and endoscopic scores in patients with CRSwNPs seems to be well established in the literature, as also confirmed by Sudiro et al. [30], whose results showed a strong positive association between radiological LMS and the modified Lund–Kennedy (MLK) endoscopic score. For these reasons, in order to perform a correct and comprehensive staging of disease severity, we recommend that CRSwNP patients should always be evaluated from endoscopic, radiologic, and laboratory viewpoints, as reported by Canevari et al. [31].

Our research showed that patients with eCRSwNPs had more sinonasal sites occupied by polyps compared to those with non-eCRSwNPs. This finding is consistent with previous studies. For instance, Fadda et al. conducted a retrospective analysis of 280 patients who had undergone ESS for CRSwNP. Their study found that eCRSwNP cases had a greater paranasal sinus extension, as radiologically determined by LMS and MLK, than non-eCRSwNP ones [19]. This, in turn, was positively associated with the endoscopic extent of the disease and the extent of surgery [30,32].

We also found that CRSwNP patients with ASA intolerance showed an increased number of sinuses involved by polyps. This finding agrees with the results of several studies that have found asthma and ASA intolerance associated with extensive patterns of CRSwNPs [32,33,34,35,36]. These conditions predispose to a spread of inflammation at the level of the airway mucosa, as supported by the fact that patients with these characteristics first develop upper airway symptoms, progress to lower airway symptoms, and finally acquire ASA intolerance [37]. This could, therefore, explain the ability of inflammation and, thus, nasal polyps to spread to a larger number of sinuses.

### 4.2. Nasal Polyps’ Recurrence

CRS is a heterogeneous disease, and according to current knowledge, it is stratified into endotypes based on different pathogenic mechanisms [7,9,10,11]. It has been demonstrated that different endotypes and molecular mechanisms are associated with various phenotypes. For this reason, the classification of CRS into endotypes is still quite difficult [38,39]. In a recent systematic review regarding protein biomarkers in patients with CRS, Gokani et al. [40] showed differences in pathophysiologic inflammatory molecular pathways in non-eCRSwNPs vs. eCRSwNPs. In the first case, the critical molecular mediators were found to be IL-1, IL-6, IL-17, and IFN-γ; in the latter, IL-4, IL-5, IL-10, and IL-13 were the leading inflammatory mediators.

According to our results, at univariate analysis, a higher risk of polyps’ recurrence was associated with the number of paranasal sinuses involved at surgery and with conditions such as asthma, ASA intolerance, high eosinophilic and basophilic blood cell percentage, and eosinophilic histotype. These same associations have been found in other recently published studies [41]. In particular, high tissue eosinophilia has been generally associated with a type 2 endotype and a more aggressive disease progression needing frequent revision ESS [21,22,42,43]. Moreover, in CRSwNPs, high tissue eosinophilia (>10 eosinophils/HPF) has been related to a higher polyps’ recurrence rate [43,44].

In this investigation, preoperative blood basophils (cell count and percentage) were related to a higher CRSwNP recurrence rate. In another recent paper by our clinical research group, blood basophil count was significantly higher in patients affected by AERD compared to controls [18]. In addition, available data have shown that specific cells may determine whether polyps will regrow by activating immunological pathways [45]. An interesting finding suggested an association between the percentage of blood eosinophils before surgery and the likelihood of relapse in patients suffering from CRSwNPs [46,47]. Our results showed that polyps’ relapse was significantly associated with the percentage of blood eosinophils rather than the actual count of these cells. A reasonable explanation for this finding could be related to the daily variability in total number of patients’ white blood cells. Sampling was not performed in the same setting as they were collected over a 12.5-year period (e.g., time of the analysis). Moreover, other associated factors, such as comorbidities or underlying immunological status, could have influenced the blood values.

As previously stated, our results, a univariate model, suggested that patients affected by eosinophilic polyposis, asthma, and ASA intolerance showed a more diffuse spreading of the disease inside nasal and paranasal cavities, associated with a higher recurrence rate. It can be hypothesized that CRSwNP in patients with eosinophilic histotype, high blood eosinophils and basophils, asthma, and ASA intolerance is intrinsically a more severe, diffuse, and aggressive disease compared to patients not affected by these conditions. This may be due to a different pathophysiological mechanism underlying polyps’ development. This speculation is based on the evidence that in our data, a higher number of patients affected by these conditions were in groups 3 and 4. In order to provide a complete understanding, additional research is required to clarify these particular aspects. Moreover, further studies may evaluate the relationship with the perceived quality of life [48].

No significant associations between CRSwNP recurrence rate and variables such as sex, age, and allergy were found. In a limited sample of 158 patients who had undergone ESS for CRSwNP, Kun Du et al. [46] had similar results regarding recurrence. However, there might be some associations between inhalant allergy and CRSwNP recurrence. Considering a large sample of 1716 patients, Tokunaga et al. [49] found significant associations between certain allergens and specific histotypes of CRSwNP.

### 4.3. Strengths and Weaknesses

The main limits of the present study are (i) the monocentric nature, (ii) the retrospective design, and (iii) the limits of patient numbers, especially in some subgroups, such as patients suffering from asthma or intolerance to ASA. Furthermore, the stratification into four groups adopted in the analysis did not consider unilateral or bilateral disease involvement inside groups and the extent of polyps inside each paranasal sinus.

The main strengths are mainly based on CRSwNP series homogeneity: (i) groups were not different in terms of age and sex; (ii) preoperative and postoperative follow-up endoscopic evaluations were performed by the same team; (iii) ESS was performed by the same surgeons; (iv) all ESS were primary surgeries; (v) histopathological analyses were performed by a dedicated head and neck pathologist; (vi) all blood tests were performed at the same laboratory; and (vii) CRSwNP relapse occurrence was always endoscopically confirmed by the same team.

## 5. Conclusions

CRSwNPs appears to be more aggressive in patients affected by conditions such as ASA intolerance, asthma, blood hyper-eosinophilia, and eosinophilic histological subtype. In the present series, these patients showed a more diffuse pattern of sinonasal polyps’ development with a higher recurrence rate. Targeted therapy with higher doses of steroids and a closer follow-up should be considered for patients suffering from these diseases. Further studies on larger series are necessary to investigate if the analysis of these characteristics in patients affected by CRSwNP could predict a possible extended development of the disease and play a role in its management. Possible future studies may be focused on identifying biomarkers or genetic factors that can predict disease extent and/or recurrence risk.

## Figures and Tables

**Table 1:** (**a**) Summary statistics of demographic, clinical data, laboratory findings, and polyps’ histology and association between groups. (**b**) Summary statistics of confidence intervals for the differences between groups.

(a)							
		Group				Total (N = 449)	*p* Value
		1 (N = 61)	2 (N = 148)	3 (N = 123)	4 (N = 117)		
**Age**							
	Mean (SD)	47.9 (16.3)	50.3 (14.2)	50.7 (13.8)	51.4 (14.6)	50.4 (14.5)	0.4905
	Median (range)	49.0 (16.0–84.0)	49.5 (18.0–83.0)	52.0 (14.0–79.0)	52.0 (17.0–84.0)	51.0 (14.0–84.0)	
**Sex**							
	Female	24 (39.3)	52 (35.1)	43 (35.0)	40 (34.2)	159 (35.4)	0.9178
	Male	37 (60.7)	96 (64.9)	80 (65.0)	77 (65.8)	290 (64.6)	
**Allergy**							
	Not present	42 (68.9)	88 (59.5)	84 (68.3)	72 (61.5)	286 (63.7)	0.3604
	Present	19 (31.1)	60 (40.5)	39 (31.7)	45 (38.5)	163 (36.3)	
**Asthma**							
	Not present	48 (78.7)	120 (81.1)	88 (71.5)	68 (58.1)	324 (72.2)	0.0003
	Present	13 (21.3)	28 (18.9)	35 (28.5)	49 (41.9)	125 (27.8)	
**ASA intolerance**							
	Not present	58 (95.1)	137 (92.6)	111 (90.2)	94 (80.3)	400 (89.1)	0.0037
	Present	3 (4.9)	11 (7.4)	12 (9.8)	23 (19.7)	49 (10.9)	
**Blood eosinophilic cell count [cell 10^9^/L]**							
	Mean (SD)	0.21 (0.15)	0.28 (0.20)	0.34 (0.26)	0.50 (0.76)	0.34 (0.44)	<0.0001
	Median (range)	0.18 (0.01–0.61)	0.22 (0.00–1.10)	0.28 (0.00–1.71)	0.35 (0.01–7.00)	0.26 (0.00–7.00)	
**Blood eosinophilic cell percentage**							
	Mean (SD)	3.17 (2.35)	4.20 (3.26)	4.96 (3.59)	6.36 (5.76)	4.83 (4.18)	<0.0001
	Median (range)	2.50 (0.10–10.40)	3.30 (0.00–23.80)	4.30 (0.00–22.40)	5.10 (0.10–49.00)	3.90 (0.00–49.00)	
**Blood basophilic cell count [cell 10^9^/L]**							
	Mean (SD)	0.030 (0.019)	0.031 (0.017)	0.036 (0.020)	0.039 (0.040)	0.034 (0.026)	0.0634
	Median (range)	0.030 (0.000–0.120)	0.030 (0.010–0.130)	0.030 (0.010–0.100)	0.030 (0.010–0.410)	0.030 (0.000–0.410)	
**Blood basophilic cell percentage**							
	Mean (SD)	0.48 (0.30)	0.46 (0.25)	0.53 (0.30)	0.56 (0.42)	0.51 (0.32)	0.0721
	Median (range)	0.40 (0.00–1.60)	0.40 (0.10–1.50)	0.50 (0.10–1.50)	0.50 (0.10–3.50)	0.50 (0.00–3.50)	
**Polyps’ histology**							
	Non-eosinophilic	50 (82.0)	90 (60.8)	61 (49.6)	41 (35.0)	242 (53.9)	<0.0001
	Eosinophilic	11 (18.0)	58 (39.2)	62 (50.4)	76 (65.0)	207 (46.1)	
(**b**)							
		**Difference (95% CI)**					
		**2 vs. 1**	**3 vs. 1**	**4 vs. 1**	**3 vs. 2**	**4 vs. 2**	**4 vs. 3**
**Age (years) (mean)**		2.4 (−2.0; 6.7)	2.7 (−1.7; 7.2)	3.5 (−1.0; 8.0)	0.4 (−3.1; 3.8)	1.1 (−2.4; 4.7)	0.8 (−2.9; 4.5)
**Female sex (%)**		4.2 (−10.3; 18.7)	4.4 (−10.5; 19.3)	5.2 (−9.8; 20.1)	0.2 (−11.2; 11.6)	1.0 (−10.6; 12.5)	0.8 (−11.3; 12.8)
**Allergy (%)**		9.4 (−4.7; 23.5)	0.6 (−13.7; 14.8)	7.3 (−7.3; 21.9)	−8.8 (−20.2; 2.6)	−2.1 (−13.9; 9.8)	6.8 (−5.3; 18.8)
**Asthma (%)**		−2.4 (−14.5; 9.7)	7.1 (−5.9; 20.2)	20.6 (7.0; 34.2)	9.5 (−0.6; 19.7)	23.0 (12.0; 33.9)	13.4 (1.5; 25.4)
**ASA intolerance (%)**		2.5 (−4.4; 9.4)	4.8 (−2.7; 12.4)	14.7 (5.7; 23.8)	2.3 (−4.4; 9.1)	12.2 (3.9; 20.6)	9.9 (1.0; 18.8)
**Eosinophilic cell count [cell 10^9^/L] (median)**		0.05 (0.01; 0.10)	0.10 (0.05; 0.16)	0.16 (0.10; 0.22)	0.05 (0.01; 0.09)	0.10 (0.06; 0.15)	0.06 (0; 0.11)
**Eosinophilic cell percentage (median)**		0.80 (0.10; 1.40)	1.50 (0.60; 2.30)	2.30 (1.50; 3.20)	0.70 (0; 1.30)	1.50 (0.80; 2.20)	0.80 (0.10; 1.60)
**Basophilic cell count [cell 10^9^/L] (median)**		0 (0; 0.01)	0 (0; 0.01)	0 (0; 0.01)	0 (0; 0.01)	0 (0; 0.01)	0 (0; 0)
**Basophilic cell percentage (median)**		0 (−0.10; 0.10)	0.10 (0; 0.10)	0.10 (0; 0.10)	0.10 (0; 0.10)	0.10 (0; 0.10)	0 (−0.10; 0.10)
**Eosinophilic polyp histology (%)**		21.2 (33.6; 8.7)	32.4 (19.3; 45.5)	46.9 (34.0; 59.9)	11.2 (−0.6; 23.1)	25.8 (14.1; 37.5)	14.6 (2.2; 26.9)

One is missing in Group 3 for blood eosinophilic and basophilic cell count and percentage.

**Table 2:** Association between recurrence-free probability and demographic, clinical data, laboratory findings, and polyps’ histology. For quantitative variables, HRs are expressed per 1 unit of increase, except for blood basophilic cell count, which is expressed per 0.01 unit of increase.

		Recurrence			Univariate Cox Regression		Multivariate Cox Regression
		No (N = 336)	Yes (N = 106)	*p* Value	HR (95 CI)	*p* Value	HR (95 CI)
**Age**							
	Mean (SD)	50.71 (14.85)	49.08 (13.24)	0.1803	0.991 (0.978–1.004)		
	Median (range)	52.00 (14.00–84.00)	47.00 (24.00–83.00)				
**Sex**							
	Female	121 (36.0)	36 (34.0)	0.5691	1		
	Male	215 (64.0)	70 (66.0)		1.124 (0.752–1.680)		
**Allergy**							
	Not present	222 (66.1)	61 (57.5)	0.0810	1		
	Present	114 (33.9)	45 (42.5)		1.409 (0.959–2.072)		
**Asthma**							
	Not present	264 (78.6)	55 (51.9)	<0.0001	1	0.0001	2.180 (1.468–0.238)
	Present	72 (21.4)	51 (48.1)		2.552 (1.743–3.738)		
**ASA intolerance**							
	Not present	312 (92.9)	81 (76.4)	<0.0001	1		
	Present	24 (7.1)	25 (23.6)		2.460 (1.569–3.856)		
**Groups**							
	1	56 (16.7)	5 (4.7)	0.0073	1		
	2	114 (33.9)	31 (29.2)		2.395 (0.931–6.161)		
	3	89 (26.5)	31 (29.2)		3.071 (1.192–7.908)		
	4	77 (22.9)	39 (36.8)		4.215 (1.658–0.713)		
**Blood eosinophilic cell count [cell 10^9^/L]**							
	N (N missing)	335 (1)	106 (0)	0.1136	1.258 (0.947–1.671)		
	Mean (SD)	0.33 (0.47)	0.40 (0.34)				
	Median (range)	0.23 (0.00–7.00)	0.32 (0.00–2.72)				
**Blood eosinophilic cell percentage**							
	N (N missing)	335 (1)	106 (0)	0.0002	1.054 (1.025–1.083)		
	Mean (SD)	4.42 (3.32)	6.23 (6.02)				
	Median (range)	3.70 (0.00–22.40)	5.15 (0.00–49.00)				
**Blood basophilic cell count [cell 10^9^/L]**							
	N (N missing)	335 (1)	106 (0)	0.0007	1.108 (1.044–1.176)		
	Mean (SD)	0.033 (0.027)	0.038 (0.023)				
	Median (range)	0.030 (0.000–0.410)	0.030 (0.010–0.130)				
**Blood basophilic cell percentage**							
	N (N missing)	335 (1)	106 (0)	0.0020	2.323 (1.361–3.962)	0.0118	2.003 (1.167–0.439)
	Mean (SD)	0.49 (0.31)	0.58 (0.36)				
	Median (range)	0.40 (0.00–3.50)	0.50 (0.10–1.90)				
**Polyps’ histology**							
	Non-eosinophilic	201 (59.8)	37 (34.9)	0.0001	1	0.0104	1.729 (1.138–0.627)
	Eosinophilic	135 (40.2)	69 (65.1)		2.207 (1.480–3.292)		

Multivariate Cox regression results only regarding the variables selected (*p* < 0.05) by the backward stepwise selection method.

## Data Availability

The data presented in this study are available on request from the corresponding author.
